# Artificial intelligence models for methylene blue removal using functionalized carbon nanotubes

**DOI:** 10.1038/s41598-023-45032-3

**Published:** 2023-10-25

**Authors:** Abd-Alkhaliq Salih Mijwel, Ali Najah Ahmed, Haitham Abdulmohsin Afan, Haiyam Mohammed Alayan, Mohsen Sherif, Ahmed Elshafie

**Affiliations:** 1 Department of Civil Engineering, College of Engineering, Universiti Tenaga Nasional (UNITEN), 43000 Kajang, Selangor Malaysia; 2 Institute of Energy Infrastructure, Universiti Tenaga Nasional (UNITEN), 43000 Kajang, Selangor, Malaysia; 3 Upper Euphrates Basin Developing Center, University of Anbar, Ramadi, Iraq; 4 Chemical Engineering Department, University of Technology, Al-Sinaa Street 52, Baghdad, 10066 Iraq; 5 National Water and Energy Center, United Arab Emirates University, P.O. Box 15551, Al Ain, United Arab Emirates; 6 Civil and Environmental Engineering Department, College of Engineering, United Arab Emirates University, 15551, Al Ain, United Arab Emirates; 7 Department of Civil Engineering, Faculty of Engineering, University of Malaya (UM), 50603 Kuala Lumpur, Malaysia

**Keywords:** Environmental sciences, Chemistry

## Abstract

This study aims to assess the practicality of utilizing artificial intelligence (AI) to replicate the adsorption capability of functionalized carbon nanotubes (CNTs) in the context of methylene blue (MB) removal. The process of generating the carbon nanotubes involved the pyrolysis of acetylene under conditions that were determined to be optimal. These conditions included a reaction temperature of 550 °C, a reaction time of 37.3 min, and a gas ratio (H_2_/C_2_H_2_) of 1.0. The experimental data pertaining to MB adsorption on CNTs was found to be extremely well-suited to the Pseudo-second-order model, as evidenced by an R2 value of 0.998, an X2 value of 5.75, a q_e_ value of 163.93 (mg/g), and a K2 value of 6.34 × 10–4 (g/mg min).The MB adsorption system exhibited the best agreement with the Langmuir model, yielding an R^2^ of 0.989, R_L_ value of 0.031, q_m_ value of 250.0 mg/g. The results of AI modelling demonstrated a remarkable performance using a recurrent neural network, achieving with the highest correlation coefficient of R^2^ = 0.9471. Additionally, the feed-forward neural network yielded a correlation coefficient of R2 = 0.9658. The modeling results hold promise for accurately predicting the adsorption capacity of CNTs, which can potentially enhance their efficiency in removing methylene blue from wastewater.

## Introduction

Nanotechnology has been recognized as a highly revolutionary technology due to its ability to unlock new possibilities in nanoscale engineering, enabling the production and utilization of materials, devices, and systems with novel features and functions^1^. Nano-adsorbents, which possess a considerable specific surface area, a diminutive intraparticle diffusion distance, and a surface that can be chemically manipulated, present a plethora of potential applications in the domain of water treatment^2^. They have the potential to introduce numerous innovative uses in the field. Due to the remarkable physiochemical characteristics of nanomaterials and the limitations of traditional adsorbents in terms of effectiveness and selectivity, carbon nanotubes (CNTs) have garnered significant attention in the industrial and scientific communities as a promising alternative from both technological and environmental perspectives^3^. To meet stringent environmental regulations, a diverse range of wastewater treatment approaches are being developed in response to the recent discharge of hazardous compounds without proper regulation^4^. Hence, there is a significant imperative to develop efficient, cost-effective, and sustainable technologies for screening and treating harmful environmental pollutants^5,6^. Adsorption has emerged as one of the most successful methods for removing a diverse range of pollutants from aqueous solutions because of its low energy requirements, ease of use, and environmental compatibility^7^. Extensive research efforts have been devoted to water quality forecasting models to enhance management plans and early warning systems^8^. Nevertheless, a persistent challenge lies in dealing with water-related data, which often involves nonlinear variables and fluctuations^9^. Researchers and developers worldwide are currently directing their attention towards artificial intelligence, particularly in the field of civil engineering. The simplicity and cost-effectiveness of AI's applications serve as its primary determinants, as they allow for precise issue estimation, handling of extensive and complex data, and solution of highly nonlinear problems that are beyond the scope of empirical equations. Due to their robustness and problem-solving capabilities, AI models have exhibited exceptional performance and superiority in processing complex nonlinear data^10^. The increasing number of published studies in recent years indicates a growing interest in utilizing the AI approach for water treatment modeling^11^. The Water Quality Index (WQI), which considers various water quality variables such as dissolved oxygen (DO), biological oxygen demand (BOD), temperature, total suspended solids (TSS), turbidity, calcium, chemical oxygen demand (COD), and pH, is widely used as a primary metric for assessing the performance of water treatment plants. Several artificial intelligence (AI) models, including artificial neural network (ANN), Multilinear Regression (MLR), Radial Basis Function (RBF), and Support Vector Machine (SVM), have been employed successfully in water quality prediction and monitoring. In many studies, neural networks (NNs) have been utilized for monitoring and assessing surface water quality^12,13^. Due to their complex chemical composition, dyes are resistant to light and oxidation, which contributes to their non-biodegradability. Consequently, the presence of dyes in water bodies, even in small quantities, can have detrimental effects on the environment^14^. One of the common ecological risks resulting from the inappropriate discharge of toxic dyes into water is the depletion of oxygen and hindered access to daylight. Methylene blue (MB) is an example of a hazardous dye. Various treatment methods, such as photochemical processes, biodegradation, electrochemical methods, synthetic coagulation, reverse osmosis, and adsorption, have been employed for azo color removal. In comparison to other physiochemical processes discussed in the literature, both human activities and the MB production process contribute to the proliferation of MB pollution in the environment^13,15^.

A review study by Bosu et al.^16^ explores the use of clay nanocomposites (CNCs) in environmental remediation of contaminants like agrochemicals and dyes. They discussed the synthesis methods, efficacy parameters, and performance assessment methods, additionally discussed machine learning applications for performance modeling and the highest sorption uptake.

Consequently, various sources of fresh water, air, landfill leachate, dust particles and wastewater were all impacted by MB exposure. The ester link that binds MB polycarbonate and resin molecules in plastic food receptacles and bottles undergoes hydrolysis upon contact with water at room temperature, leading to the leakage of MB monomer. Dyes are extensively acknowledged as substances that have the potential to cause cancer and genetic mutations, resulting in a range of detrimental impacts on human well-being. The immediate exposure to methylene blue can give rise to profound health issues, encompassing impairment of the cognitive faculties, the nervous system, the renal system, the hepatic system, and the reproductive system. Methylene blue can also cause skin photosensitization, resulting in a bluish coloration. Inhalation of methylene blue can cause difficulty in breathing, while inadvertent ingestion may lead to a burning sensation, along with symptoms such as nausea, diarrhea, vomiting, and gastritis. The intricate molecular composition of methylene blue contributes to its resistance to light, oxidation, traditional biological and physical oxidation treatments, and amplifies its non-biodegradable characteristics. The inappropriate discharge of methylene blue (MB) into natural bodies of water presents notable environmental hazards as it diminishes oxygen levels and hampers sunlight penetration, thus adversely impacting photosynthesis activity in aquatic plankton^17^. The objective of this study is to minimize the costs associated with isolation and enhance the adsorbent capacity by fabricating a novel type of hybrid carbon nanotubes (CNTs) on a substrate of powdered activated carbon (PAC), resulting in the development of multi-structured materials spanning from the nano to micro scales. The prepared hybrid material exhibits chemical homogeneity due to its primarily carbon composition, while also possessing a heterogeneous structure with multiscale particles of varying shapes.

## Research objectives

This study aims to achieve the following obejctives:

1. To investigate the impacts of pH, the quantity of adsorbent used, and the duration of contact on the efficiency of adsorption and the experimental efficacy of methylene blue elimination, we conducted a study utilizing artificially engineered carbon nanotubes(CNTs)^17^.
2. To explore the kinetics and isotherm properties of various adsorbate-adsorbent systems under different circumstances.
3. To assess the feasibility of employing an artificial intelligence model to simulate the MB removal ability of synthetic carbon nanotubes.
4. To develop multiple prediction models for methylene blue elimination.

## Methodology

**Figure 1:**
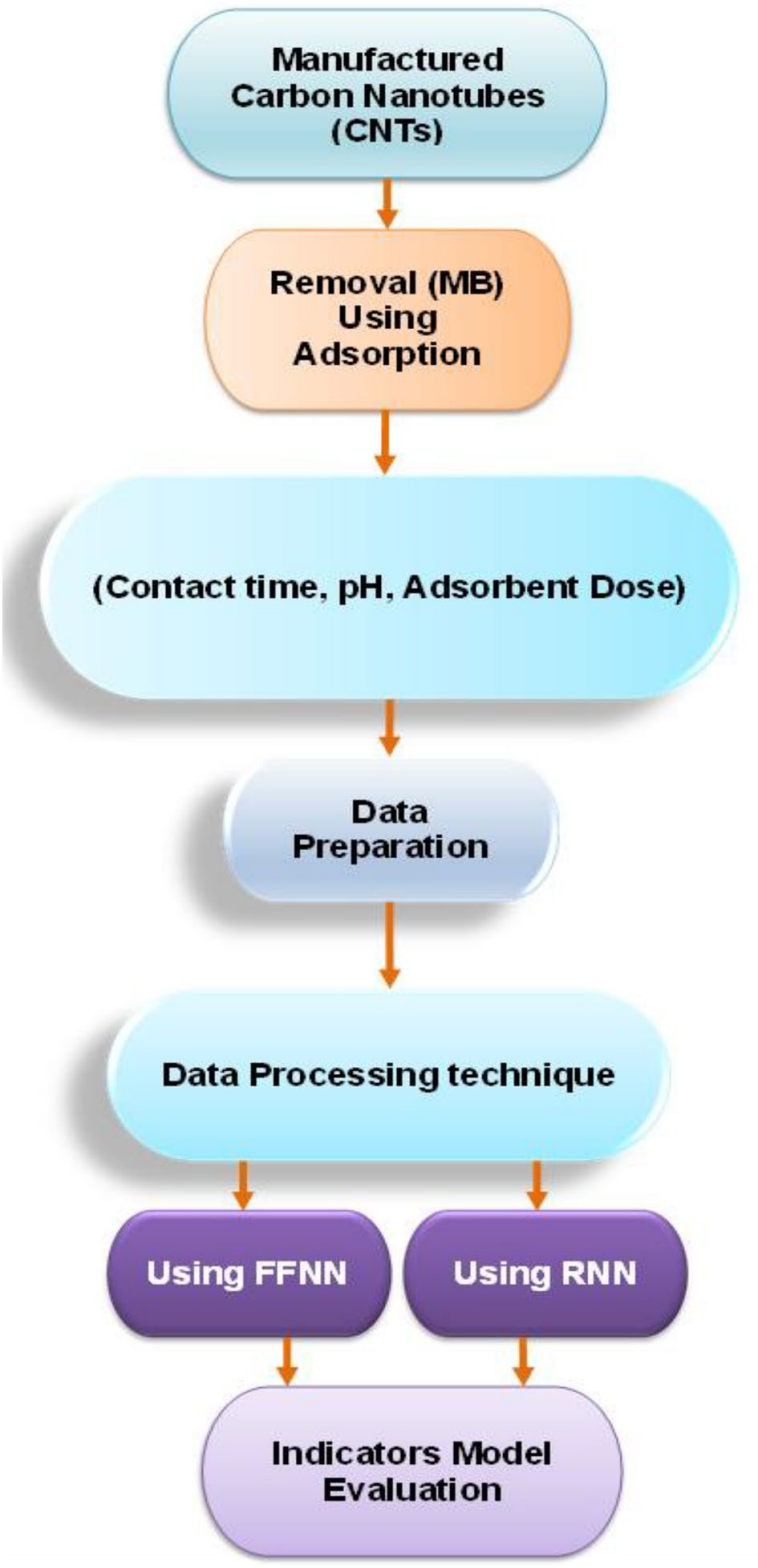
Methodology flowchart.

The present study's methodology is bifurcated into two segments. The initial segment pertains to the compilation of data, with a specific focus on chemical endeavors. The second segments involves developing the artificial intelligence model. The flowchart (Fig. 1) illustrates the steps of the methodology of the study, starting from the manufacturing of CNTs and extending to the evaluation of AI models. Each step is discussed in detail in the following section.

### Materials and chemicals

The study includes a list of reagents and chemicals utilized in this study, along with their suppliers, purity grade, and applications. The general properties of Methylene blue are presented in Table 1.

**Table 1:** The general characteristics and chemical composition of (Methylene blue)^17^.

Characteristics	Values
Molecular formula	C16H18 ClN3S
Molecular weight (g/mol)	319.8
λ max (nm)	665
Chemical structure	
Space-filling model	

### Batch adsorption experiments

In order to ascertain the adsorption propensity of carbon nanotubes (CNTs) in the context of eliminating Methylene blue (MB) from water, a series of mass adsorption experiments were carried out. Three investigations were carried out, including batch tests with MB pollutants, kinetic studies, and isotherm studies. MB concentrations were measured at 665 nm utilizing a UV–visible spectrophotometer. The adsorption experiments were performed in 250 mL Erlenmeyer flasks with glass stoppers. The required amount of adsorbate (MB) was dissolved in a 1000 mL volumetric flask, and deionized water was added to reach the mark, producing the stock solution of the adsorbate (MB). Batch adsorption tests were conducted on the carbon nanotubes (CNTs). Each adsorbent was added at a fixed dose of 10 mg per 50 mL of contaminant (50 mg/L). The mixture was stirred at a constant speed of 180 rpm for 120 min at room temperature, maintaining a pH of 6.0. After the period of adsorption, a specified quantity of the solution was extracted and subjected to centrifugation at a rate of 4000 revolutions per minute for a duration of 10 min. The content of the sorbate in the resulting liquid above the sediment was estimated by observing the wavelength at which the absorbance reached its maximum using a spectrophotometer that operates in the ultraviolet–visible range. Following this, the efficiency of removal was determined by utilizing Equation to calculate the percentage (1).

1 $$Removal\left( \% \right) \, = \, \left( {{\text{C}}_{{\text{o}}} - {\text{C}}_{{\text{t}}} } \right)/{\text{C}}_{{\text{o}}}$$

whereas q_e_ (mg/g), Using Equation, the equilibrium contact time adsorbate concentration was calculated 2.

2 $${\text{q}}_{{\text{e}}} = \left( {{\text{C}}_{{\text{o}}} {-}{\text{C}}_{{\text{e}}} } \right){\text{ V}}/{\text{w}}$$

where C_o_, C_t_, and C_e_ (mg/L) represent the initial liquid-phase adsorbate concentration, adsorbate concentration at time t (min), and adsorbate concentration at equilibrium time, respectively. While $V$ is presenting the volume of the solution. (in liters), $w$ is presenting the mas for adsorbent, (in grams). Table 2 provides a list of the adsorption parameters.

**Table 2:** Summary for parameters of MB on CNTs^17^.

Factor	Name	units	Low	High
1	pH		2	11
2	Dose	$\mathrm{mg}$	5	20
3	Contact time	$\mathrm{min}$	10	120

The empirical observations were conformed to various isotherm frameworks, such as Langmuir, Freundlich, and Temkin, in order to ascertain the process of adsorption. The initial concentration of MB employed in the kinetic assessments was 50 mg/L, and this value was consistently upheld throughout the entirety of the investigations. Kinetic modeling was employed to predict the appropriate rate expressions for reaction mechanisms and estimate the rate of contaminant removal from aqueous effluents through sorption. Similar to batch equilibrium studies, kinetic parameters were evaluated, and different contact times were employed to assess the applicability of the investigated kinetic models^18^. The optimal kinetic models that best matched the experimental data were selected based on error functions, including the nonlinear chi-square (X^2^) and the linear coefficient of determination(R^2^)^19^.

### Artificial intelligence models

The methodology of the proposed study will be based on the application of artificial intelligence (AI) models. This study seeks to assess the efficacy of AI models through the comparison of the results obtained from two fundamental models, specifically the feedforward neural network and the recurrent neural network. The adsorption capacity of functionalized carbon nanotubes in aqueous solutions will be predicted using MATLAB's NN Toolbox R2014a. The independent variables in the experiment are pH, absorbent dosage, and contact time. Artificial neural networks (ANNs) are advanced statistical techniques utilized in this study. The method employed in this research involved creating a logical model consisting of interconnected neurons in a computer network that emulates the functioning of the human nervous system. Neural networks are utilized for tackling complex test models involving tasks such as pattern recognition, classification, and estimation^20–23^. There are two types of artificial neural networks (ANNs): supervised and unsupervised. Supervised ANNs are used for classification tasks, while unsupervised ANNs are used for regression tasks^24,25^. In the supervised model, the network is educated using annotated data to modify the optimal weight values across neurons, thus enabling it to produce the intended output value(s) upon encountering novel input data. In contrast, the unsupervised model does not have a specific target output value when provided with input data. For this study, the supervised technique was employed. To produce multiple data sets for testing and training the ANN model, the prepared data were divided into specific percentages. However, the division was structured such that the majority of the data constituted the training set. The data was then rearranged within the spreadsheet and examined to ensure the absence of any pre-existing combinations of trends or inherent characteristics within the data. In order to analyze layer recurrence and feed-forward backpropagation (BP) in the RNN model, several factors were taken into account, including the number of neurons, layers, testing and training sets, and the choice of transfer function. The connection weights, denoted as WI, link the input to the hidden layer.

For both the RNN and FFNN models, the weights and biases were initialized to zero and then modified iteratively using the stochastic gradient descent (SGD) optimizer, which employed a learning rate of 0.01.

#### Feedforward neural network

**Figure 2:**
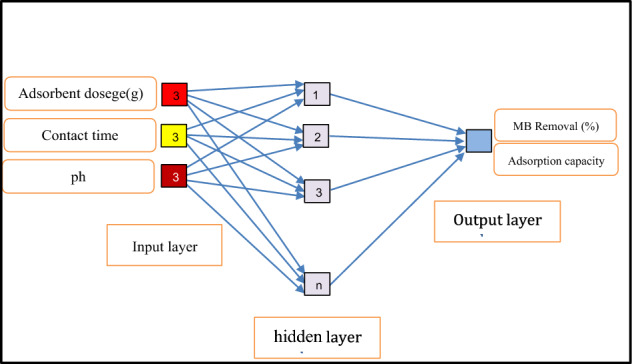
FFNN model structure.

The feedforward neural network (FFNN) is widely recognized as one of the earliest and most influential algorithms in the field of machine learning (ML). It is also known as a multilayer perceptron (MLP) or simply a neural network (NN). The FFNN structure comprises three tiers of neurons: the initial tier, one or more concealed tiers, and the terminal tier. Every neuron in a specific tier is linked to neurons in additional tiers via weighted connections (w). Neurons can be described as mathematical expressions that process information within the network. The input layer receives information in the form of input parameters, which are subsequently passed on to the next layer, called the hidden layer(s). The hidden layer(s) serves as a crucial component in connecting the input and output layers, facilitating the transformation between these two layers. It comprises multiple neurons responsible for carrying out the necessary computations. Each neuron is linked to other neurons through weighted connections, which quantify the strength of the connections. The output layer represents the target of our study, as it is the layer from which we seek to make predictions. The overall process of the FFNN can be summarized as follows: Firstly, each input parameter in the input layer is multiplied by its corresponding weight, and then bias is added to each product obtained in the previous step. This helps adjust the inputs to more practical and meaningful ranges. Subsequently, activation functions are applied to map the features between the input and output layers. Finally, by aggregating the results obtained for each neuron in the previous steps, the desired outputs are achieved. Figure 2 provides a simple illustration of the model structure for the FFNN, showcasing its input and output variables.

#### Recurrent neural network

**Figure 3:**
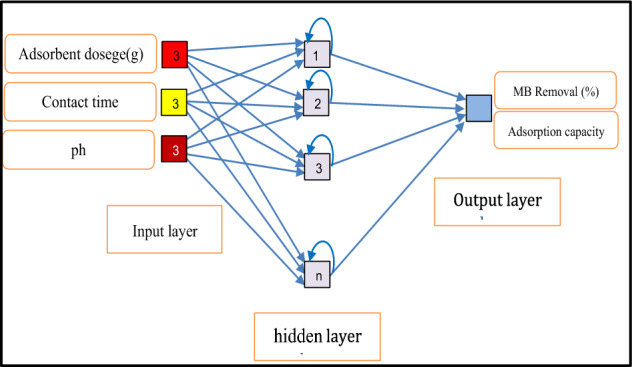
RNN model structure.

This subsection provides a basic overview of recurrent networks without delving into the specifics of the technique. For training, these networks often utilize a form of backpropagation. Many hydrologic systems demonstrate geographical and temporal variability, requiring a dynamic estimation approach. Appropriately selected artificial neural networks can effectively simulate such dynamic interactions. In the simplest scenario, a node computes the cumulative weighted sum of its inputs after being processed by a nonlinear activation function. Figure 3 depicts the model structure of an RNN, including the input and output parameters.

Recurrent backpropagation is a neural network approach that can be employed with networks featuring arbitrary connections^26^. Recurrent back-propagation is briefly described by The technique is briefly outlined by^27^, including its mathematical properties and implementation details.

#### Performance criteria

Two competing neural networks, FFNN and RNN, were devised for the development of the ANN model in this investigation. Two neural network models, FFNN and RNN, were developed and utilized in this study to construct the ANN model. The variables investigated in the experiment included adsorbent dosage, pH, and contact time. Multiple criteria were evaluated to assess the effectiveness of the FFNN and RNN models. The comparison between actual and simulated data was conducted to determine the performance of each model. The evaluation metrics used for assessing the simulation performance of the models included RE (relative error), MAPE (mean absolute percentage error), RMSE (root mean square error), MSE (mean square error) and RRMSE (relative root mean square error). The abbreviations RRMSE, MSE, RMSE, MAPE, and RE represent their respective evaluation metrics.

3 $${\text{ RRMSE}} = \left[ {\frac{1}{{\text{n}}}\mathop \sum \limits_{{{\text{t}} = 1}}^{{\text{n}}} \left( {\frac{{{\text{D}}_{{{\text{a}}\left( {\text{t}} \right)}} - {\text{D}}_{{{\text{f}}\left( {\text{t}} \right)}} }}{{{\text{D}}_{{{\text{a}}\left( {\text{t}} \right)}} }}} \right)^{2} } \right]^{\frac{1}{2}}$$

4 $${\text{MSE}} = \frac{1}{{\text{n}}}\mathop \sum \limits_{{{\text{t}} = 1}}^{{\text{n}}} \left( {{\text{D}}_{{{\text{a}}\left( {\text{t}} \right)}} - {\text{D}}_{{{\text{f}}\left( {\text{t}} \right)}} } \right)^{2}$$

5 $${\text{RMSE}} = \left[ {\frac{1}{{\text{n}}}\mathop \sum \limits_{{{\text{t}} = 1}}^{{\text{n}}} \left( {{\text{D}}_{{{\text{a}}\left( {\text{t}} \right)}} - {\text{D}}_{{{\text{f}}\left( {\text{t}} \right)}} } \right)^{2} } \right]^{\frac{1}{2}}$$

6 $${\text{MAPE}} = \frac{1}{{\text{n}}}\mathop \sum \limits_{{{\text{t}} = 1}}^{{\text{n}}} \left| {\frac{{\left( {{\text{D}}_{{{\text{a}}\left( {\text{t}} \right)}} - {\text{D}}_{{{\text{f}}\left( {\text{t}} \right)}} } \right)}}{{D_{a\left( t \right)} }}} \right| \times 100$$

7 $${\text{RE}} = \frac{{{\text{D}}_{{{\text{a}}\left( {\text{t}} \right)}} - {\text{D}}_{{{\text{f}}\left( {\text{t}} \right)}} }}{{{\text{D}}_{{{\text{a}}\left( {\text{t}} \right)}} }} \times 100$$

where one-quarter of the exact value of Da(t) ${\mathrm{D}}_{\mathrm{f}\left(\mathrm{t}\right)}$ is equivalent to the computed value, SSres ¼ represents the sum of squares for regression, while SStot symbolizes the sum of the squares of residuals. RRMSE, MSE, RMSE, MAPE, and RE serve as the metrics utilized to assess the efficacy of the model. Multiple metrics are employed to ascertain the precision of the model. These metrics are derived through the comparison of disparities between the actual and predicted outcomes.

## Findings and discussion

This section presents the findings of the study and provides a comprehensive discussion of the results.

### FESEM and TEM analyses

**Figure 4:**
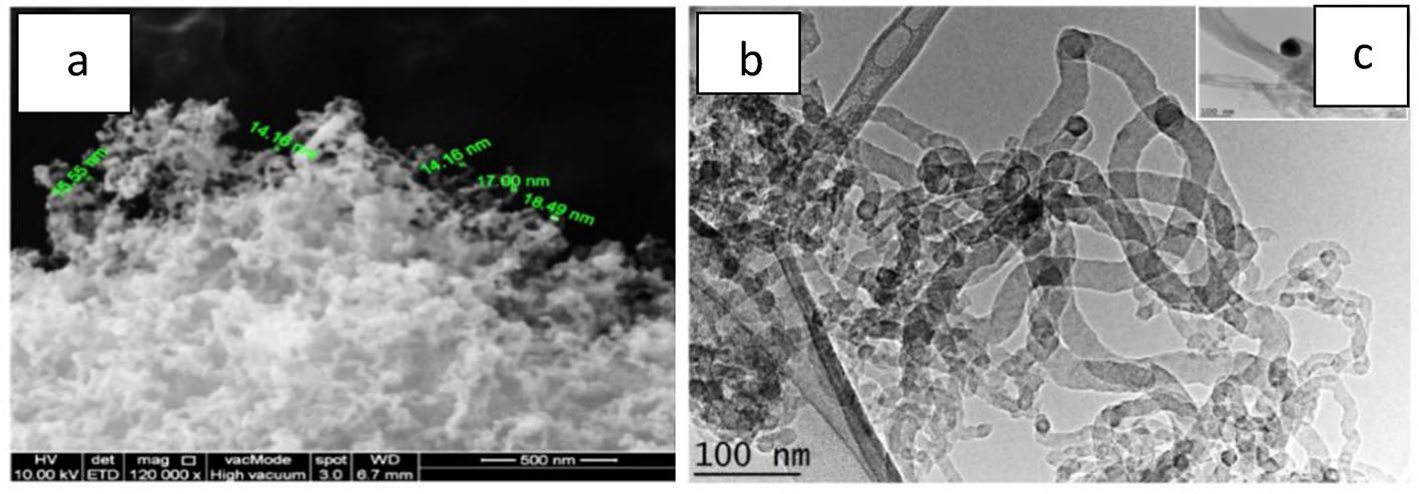
FESEM and TEM images of CNT.

This section is dedicated to the analysis of the synthesized CNTs' characterization. The morphology of the synthesized CNTs is depicted in Fig. 1 through the FESEM and TEM images. Upon microscopic analysis, it was found that the synthesized CNTs predominantly consisted of dense CNTs with tubular structures, as seen in Fig. 4a. The TEM image in Fig. 4b revealed CNTs that were well-graphitized and had an outer diameter ranging from 10 to 40 nm. It is noteworthy that these CNTs exhibited a closed tip, which was tilted from the vertical direction and originated from Ni particles. The Ni particles had an average diameter size of 70 nm. The presence of catalytic particle encapsulation at the tip, as shown in Fig. 4c, indicated that the growth of CNTs followed the tip growth mechanism. These observations differ from the findings of previous studies, which resulted in the production of a singular type of CNF^28^.

### Adsorption isotherms

**Figure 5:**
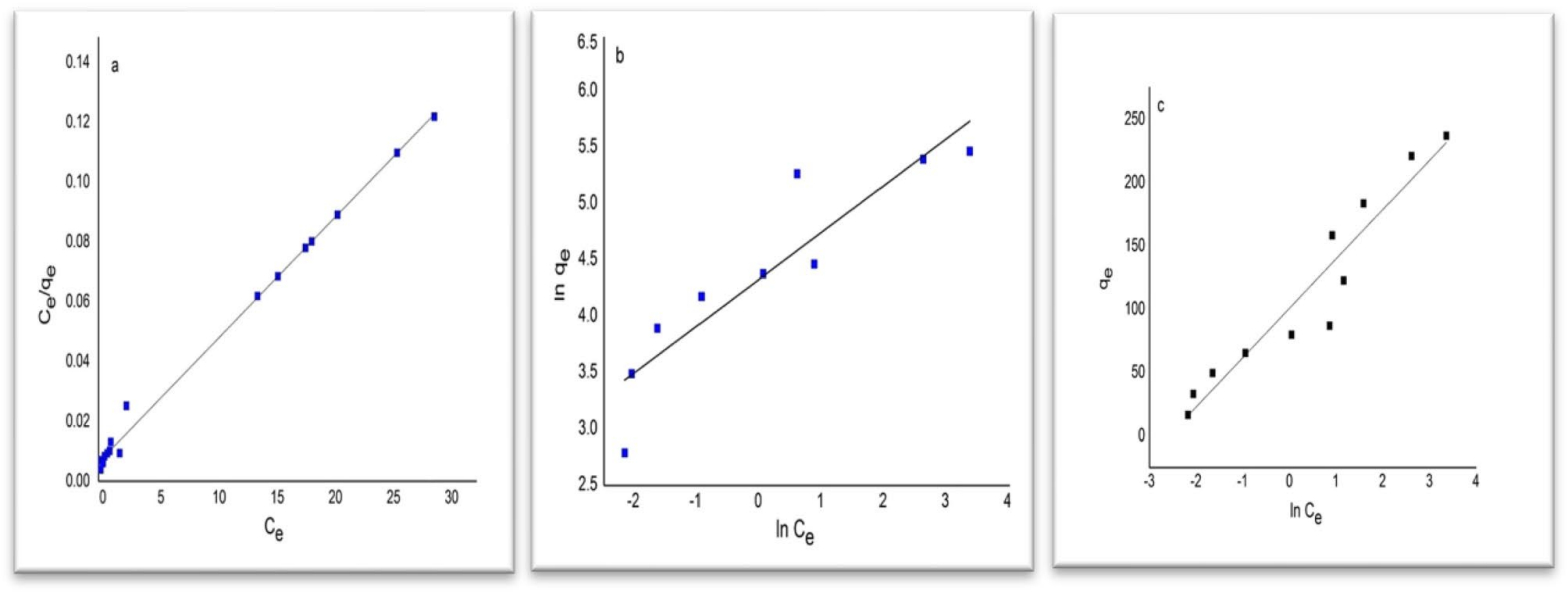
The isotherm graphs for MB adsorption on CNTs based on the data presented in the (**a**), (**b**), and (**c**) Langmuir, Freundlich, and Temkin models^29^.

As depicted in Fig. 5, the equilibrium adsorption data were assessed using the Langmuir, Freundlich, and Temkin models, as denoted by (a), (b), and (c) respectively. Table 3 illustrates the linearized equations and associated parameters for these models. Within the experimental conditions, the Freundlich isotherm indicates a favorable adsorption of MB onto CNT, as suggested by the values of the Freundlich constants (RL = 0.031 and n = 2.8). Conversely, the Langmuir isotherm exhibited the highest correlation coefficient and best fit (R2 = 0.989), with a maximum adsorption capacity of 250 mg/g. This implies that monolayer MB adsorption transpires on the uniform surface of the prepared adsorbent. An analogous equilibrium outcome was observed in the adsorption of MB onto an economical bio-waste sorbent. The Langmuir model demonstrated the lowest standard deviation, signifying a close concurrence with the experimental findings. Thus, it can be contended that the Langmuir isotherm offers the most accurate depiction of MB adsorption on the surface of CNT.

**Table 3:** Equations describing the investigated isotherm models for MB adsorption on carbon nanotubes^28^.

Model	Equation	Parameters	Values
Langmuir	$\frac{{C}_{e}}{{q}_{e}}= \frac{1}{{K}_{L} {q}_{m}} +\left(\frac{1}{{q}_{m}}\right){C}_{e}$	Q_m_	250.0
		KL	0.645
		R2	0.989
		RL	0.031
		S.D %	13.80
Freundlich	$ln{q}_{e}=ln{K}_{f }+ \frac{1}{n} ln{C}_{e}$	R2	0.855
		Kf	85.038
		N	2.832
		S.D %	18.89
Temkin	${q}_{e}= {B}_{1}ln{K}_{T} +{B}_{1}ln{C}_{e}$	S.D %	25.69
		KT	2.695
		B1	39.401
		R2	0.859

### Adsorption kinetics

**Figure 6:**
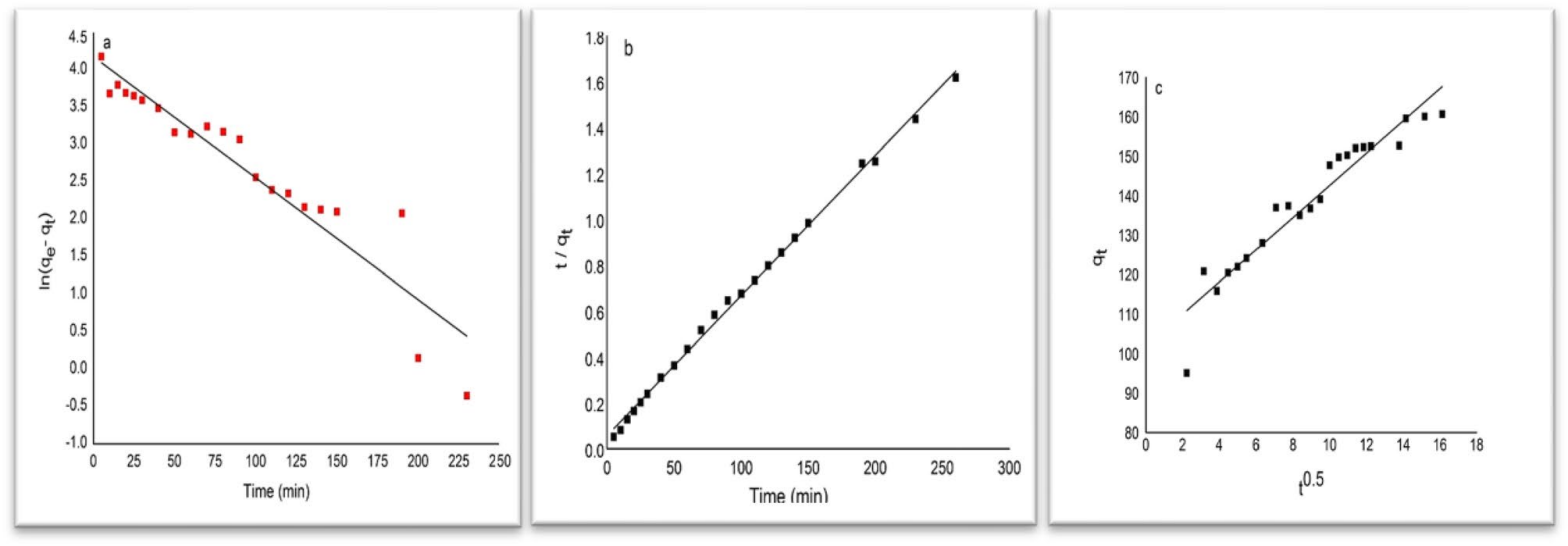
(**a**) Pseudo-first order, (**b**) Pseudo-second order, and (**c**) Intraparticle diffusion.

The data obtained from the experiment were subjected to fitting procedures according to different kinetic equations. The distinctive parameters of each of these models, including the linear coefficient of determination (R2) and non-linear Chi-square (X2), were consolidated and presented in Table 4. The illustrations of the analyzed dynamic models can be observed in Fig. 6. A considerable R2 value and a diminutive X2 value signify a commendable concordance between the dynamic model and the empirical information^28^. As stipulated in Table 4, the pseudo-second-order kinetic model offers the most proficient elucidation for the adsorption of MB onto CNT, as it showcases the utmost correlation coefficient. This is supported by the smallest R2 and X2 values (0.988 and 5.75, respectively) compared to other models. Therefore, the MB adsorption onto the CNT adsorbent follows the pseudo-second-order kinetics model, which precisely describes the system's behavior. This observation is consistent with previous findings on the MB adsorption kinetics of carbon dioxide adsorbents^17,29,30^. The chemical sorption that takes place during the adsorption of MB onto CNT is considered to be the rate-controlling phase, as per the Pseudo-second order model. This sorption involves valence forces that arise from the sharing or exchanging of electrons between the pigment and the adsorbent^31–33^. Additionally, Fig. 6c displays a relatively linear graph acquired through the regression analysis of qt against t from the regression analysis of qt versus t0.5, which yields an R^2^ value of 0.914. However, the disparity between the line and the origin implies that external mass transfer could potentially play a significant role in the adsorption process, in addition to intraparticle diffusion^28,31^. The observation is supported by the noteworthy intercepts witnessed in the linear segment of the graph (C = 101.79), which signifies a notable involvement of the CNT surface in the removal of MB and emphasizes the significance of diffusion in the boundary layer^33^. A comparison of the utmost adsorption capacity of MB on different adsorbents is exhibited in Table 5.

**Table 4:** Linearized equations of investigated kinetic models for MB adsorption on activated carbon nanotubes CNT^31^.

Model	Equation	Parameters	Values
Pseudo-first-order	$ln\left({q}_{e}-{q}_{t}\right)=ln{q}_{e}-{K}_{1}t$	R^2^	0.884
		X^2^	16.91
		K	0.012
		q_e_	51.79
Pseudo-Second-Oder	$\frac{\mathrm{t}}{ {\mathrm{q}}_{\mathrm{t}}}=\frac{1}{{\mathrm{K}}_{2}{\mathrm{q}}_{\mathrm{e}}^{2}}+\frac{1}{{\mathrm{q}}_{\mathrm{e}}}\mathrm{t}$	R^2^	0.988
		X^2^	5.75
		K_2_	6.34 × 10^–4^
		q_e_	163.93
Intraparticle diffusion	${\mathrm{q}}_{\mathrm{t}}={\mathrm{K}}_{\mathrm{d}}{\mathrm{t}}^\frac{1}{2}+\mathrm{C}$	R^2^	0.914
		X^2^	7.1
		K_d_	4.08
		C	101.79
q_e_ (experimental) = 166.11 mg/g

**Table 5:** Comparison of the maximal adsorption capacity (q_m_) for MB removal between CNT and other reported adsorbents.

Adsorbent	qm (mg/g)	References
CNM-PAC	250	The present work
CA-APT	207.48	^34^
MMT@C nanocomposites	194.2	^35^
Attapulgite / bentonite (50%)	168.63	^36^
Titanate nanotubes	133.33	^33^
Activated carbon/NiFe2O4	182.82	^37^
Powdered activated carbon	91.0	^38^
Oxidized MWCNTs	188.68	^29^
CNTs from acetylene cracking	35.4–64.7	^39^
Activated carbon/OPW	90.1	^40^
Luffa cylindrica fibers	49.0	^41^
Palygorskite	50.80	^42^
halloysite nanotubes (HNTs)	84.32	^43^

**Figure 7:**
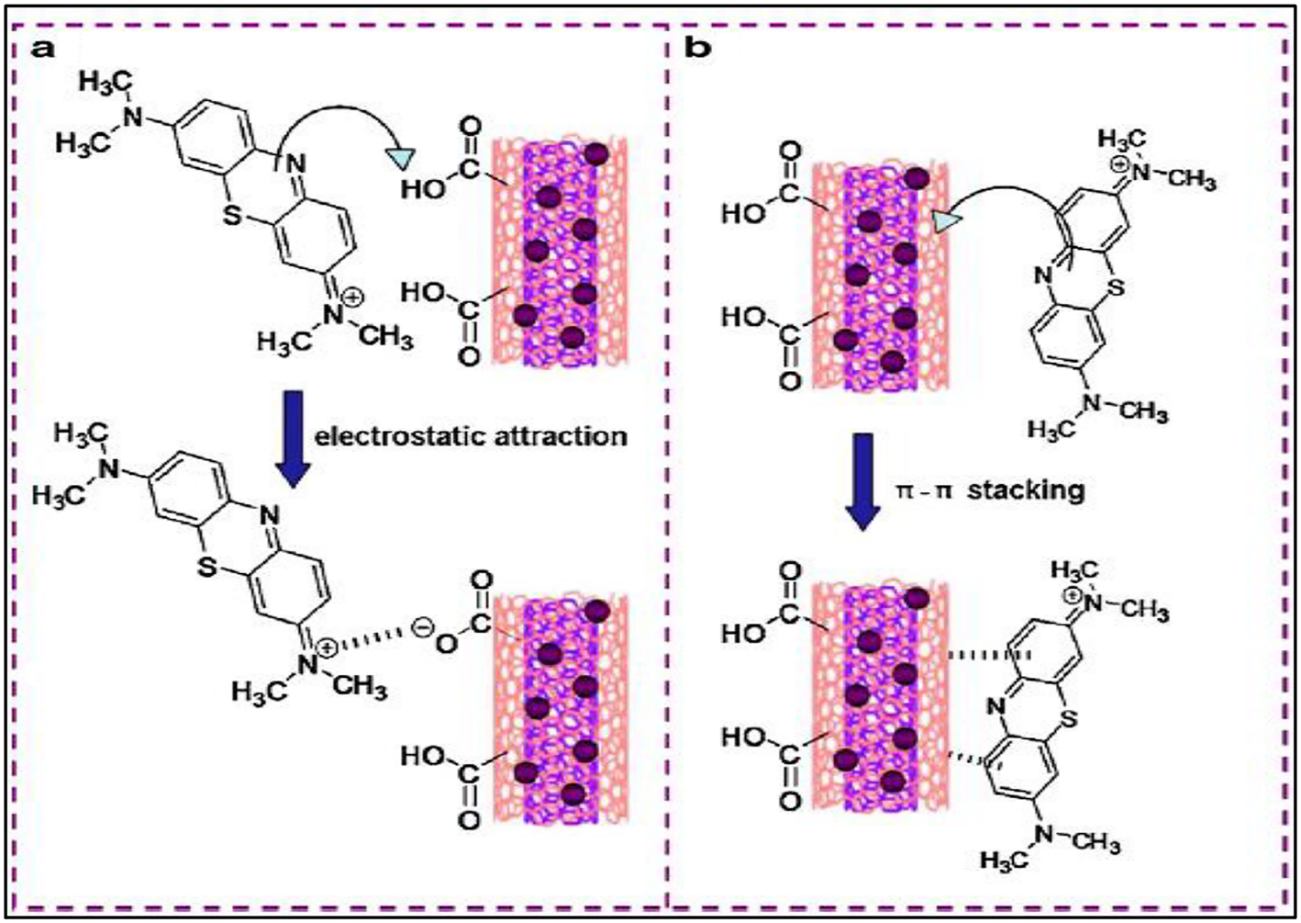
Schematic illustration of the possible interaction between MWCNTs and methylene blue: (**a**) electrostatic attraction and (**b**) π–π stacking.

**Figure 8:**
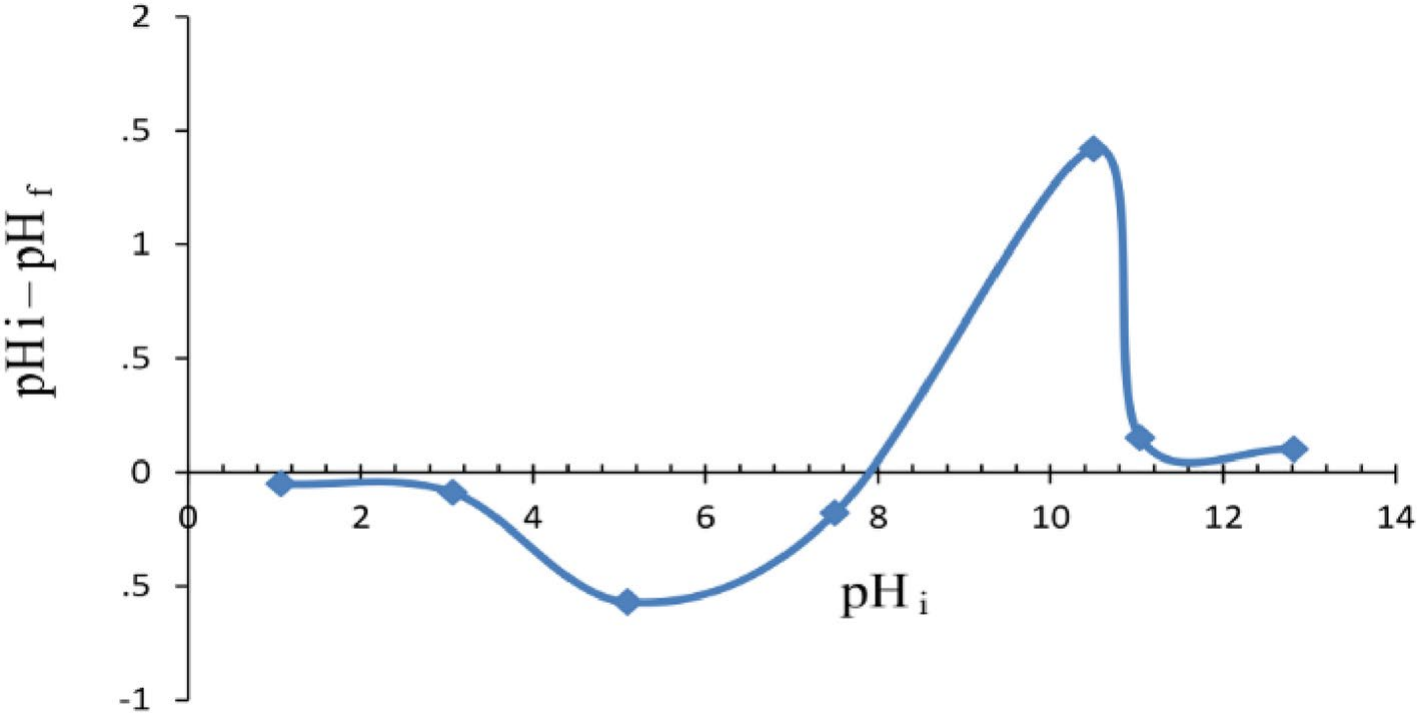
Determination of the point of zero charge of the CNTs by the pH drift.

Carbon nanotubes (CNTs) are considered to be suitable candidates as adsorbents for the pre-concentration and elimination of pollutants from large volumes of wastewater. The comprehensive findings derived from the investigation propose that the primary mechanism of adsorption for both cationic and anionic dyes on carbon nanomaterials (CNMs) is attributed to the interaction of the electron donors (such as highly polarizable graphene sheets) with the electron acceptors (aromatic molecules) present in carbon nanomaterials. Furthermore, there is a strong occurrence of surface complexation between ions and functional groups that are present on the CNMs, as depicted in Fig. 7. Furthermore, the higher MB adsorption under basic condition may be attributed to the electrostatic attraction between the cationic species of MB with the negatively charged surfaces. The surface charge assessed by the point of zero charge (pH_PZC_) is defined as the point where the zeta potential is zero. When pH < pH_PZC_, the surface charge is positive, and when pH > pH_PZC_, the surface charge is negative. In this case, the pH_PZC_ of CNTs determined by the pH drift method is about 8.0 (see Fig. 8).

### FFNN modeling and performance

The data was modeled using artificial neural networks. The performance of each model was assessed using indicators such as RRMSE, MSE, MAPE, RMSE, and RE%. These indicators were compared among the models, and the one with the lowest values was considered the optimal model. During the process of data validation, the number of neurons in the concealed layer was altered within the range of 3 to 12. The most effective number of neurons was ascertained by evaluating the minimal values of RRMSE, MSE, MAPE, RMSE, and RE%, in conjunction with the maximal value of R2. The outcomes of this particular selection process can be observed within Tables 6 and 7.

**Table 6:** The performance indicators for FFNN models to predict R1.

Model	Neurons	Structure	RRMSE	MSE	MAPE	RMSE	RE%	R^2^
M1	3	(3-3-1)	0.523	1081.72	39.94	32.88	125.10	0.0617
M2	4	(3-4-1)	0.463	1382.90	43.12	37.18	−61.87	0.0321
M3	5	(3-5-1)	0.045	14.54	3.37	3.81	−10.20	0.9658
M4	6	(3-6-1)	0.648	703.93	40.15	26.53	180.68	0.153
M5	7	(3-7-1)	0.081	32.86	4.417	5.73	23.12	0.9297
M6	8	(3-8-1)	0.458	343.84	24.99	18.54	117.81	0.2543
M7	9	(3-9-1)	0.152	87.61	9.06	9.36	43.67	0.7874
M8	10	(3-10-1)	0.226	271.53	18.74	16.47	−48.86	0.5474
M9	11	(3-11-1)	0.523	439.38	26.04	20.96	189.78	0.2038
M10	12	(3-12-1)	0.507	755.14	33.84	27.47	146.02	0.006

**Table 7:** The performance indicators for FFNN models to predict R2.

Model	Neurons	Structure	RRMSE	MSE	MAPE	RMSE	RE%	R^2^
M1	3	(3-3-1)	0.152	647.05	11.09	25.43	41.21	0.776
M2	4	(3-4-1)	0.130	597.58	11.02	24.43	22.11	0.7862
M3	5	(3-5-1)	0.162	609.55	12.40	24.68	41.76	0.8065
M4	6	(3-6-1)	0.132	454.60	7.40	21.32	42.11	0.836
M5	7	(3-7-1)	0.303	3200.20	25.50	56.57	−51.48	0.6835
M6	8	(3-8-1)	0.391	4720.98	30.73	68.70	87.07	0.4568
M7	9	(3-9-1)	0.223	2574.11	13.42	50.73	60.94	0.1693
M8	10	(3-10-1)	0.185	1414.75	17.63	37.61	−28.24	0.6492
M9	11	(3-11-1)	0.173	839.35	14.55	28.97	34.37	0.7315
M10	12	(3-12-1)	0.660	10,770.53	46.69	103.78	196.60	0.2837

**Figure 9:**
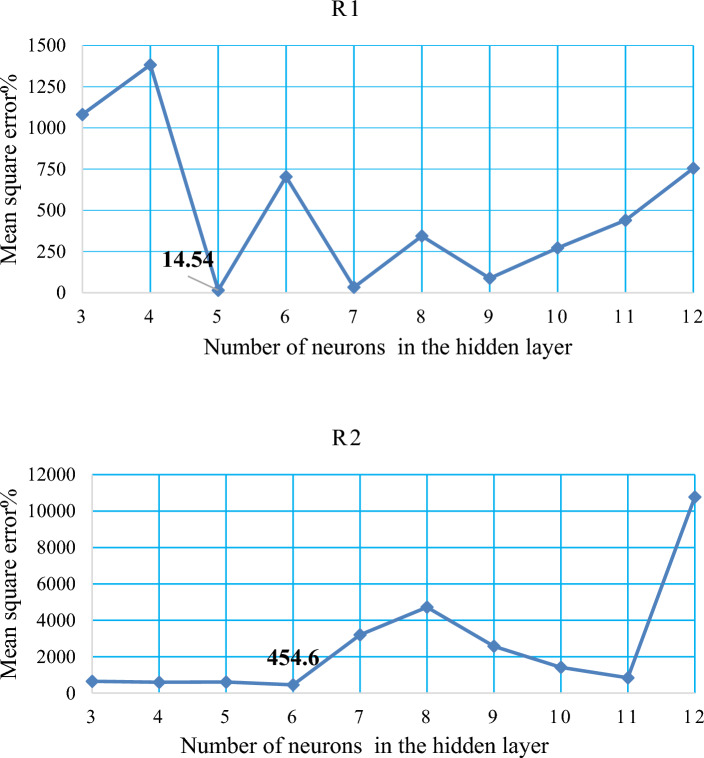
Show the correlation between the number of concealed layer neurons and the MSE obtained to predict (R2, R1).

The MSE value was observed to be 1081.72 for 3 neurons in the hidden layer. However, the MSE increased to 1382.90 when 4 neurons were utilized. Interestingly, the application of 5 hidden neurons resulted in a significant decrease in the MSE to 14.54, indicating a more stable network. This trend is illustrated in Fig. 9R1. Subsequently, with the implementation of 6 hidden neurons, the MSE value sharply increased to 703.93. For 7 neurons, the MSE decreased to 32.86, but it significantly increased to 343.84 with 8 neurons in the hidden layer. However, when 9 neurons were employed, the MSE decreased to 87.61. Finally, with the application of 10, 11, and 12 hidden neurons, the MSE values displayed a gradual increment to 271.53, 439.38, and 755.14, respectively.

The MSE value was found to be 647.05 for 3 neurons in the hidden layer. However, the MSE decreased to 597.58 when 4 neurons were utilized. With the application of 5 hidden neurons, the MSE increased to 609.55. Interestingly, the implementation of 6 hidden neurons resulted in a lower MSE value of 454.60, indicating a more stable network. This is depicted in Fig. 9R2. However, when 7 and 8 hidden neurons were used, the MSE values significantly increased to 3200.20 and 4720.98, respectively. On the other hand, with 9 neurons in the hidden layer, the MSE decreased to 2574.11. Further improvement was observed with 10 neurons, resulting in an MSE of 1414.75, and with 11 neurons, resulting in an MSE of 839.35. However, with the application of 12 hidden neurons, the MSE value increased to 10,770.53.

**Figure 10:**
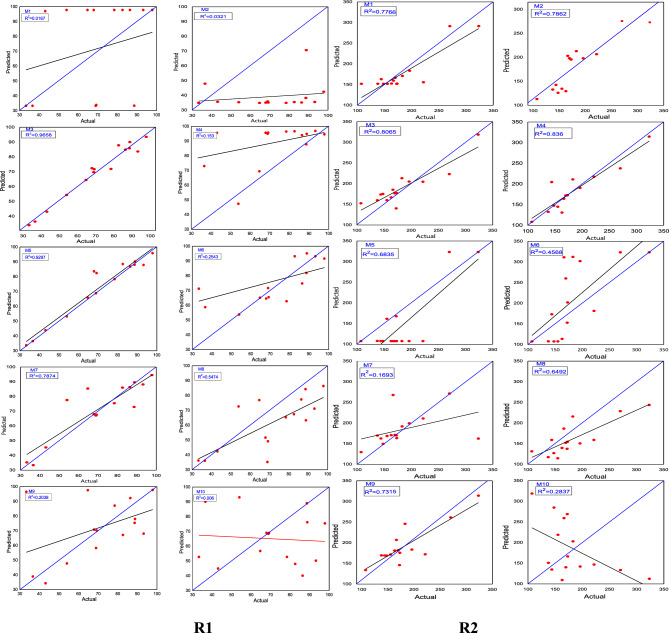
The correlation coefficient between the actual and the predicted values, presented for (R1, R2).

The scatter plots comparing the FFNN data with the experimental data were based on selecting the model with the minimum MSE value and the maximum correlation coefficient (R^2^). The model with the lowest MSE value of 14.54 and a high correlation coefficient (R^2^) of 0.9658 is considered the best model for predicting R1, as depicted in Fig. 10R1.

Similarly, for predicting R^2^, the model with the minimum MSE value of 454.60 and the maximum correlation coefficient (R2) of 0.836 is considered the best model. This model demonstrates a strong correlation between the actual and predicted values, as shown in Fig. 10R2.

**Figure 11:**
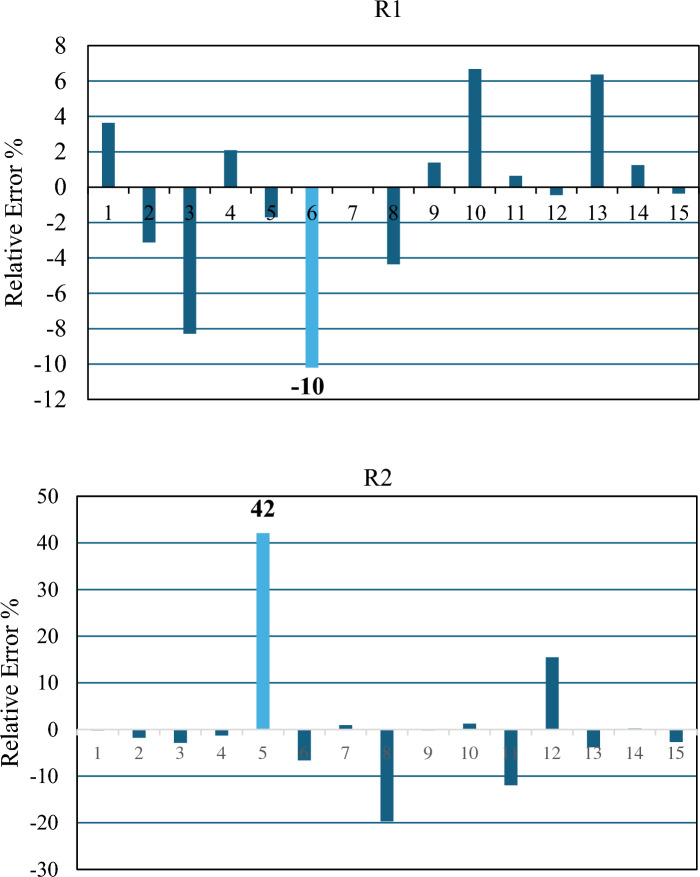
illustrates the model's precision of R1, R2.

Among the indicators used to assess the accuracy of the predicted values by the model, the relative error stands out. By conducting measurements and making comparisons between the anticipated values and the real values, one can assess the calculations in relation to their precision and accuracy. Accuracy denotes the degree to which the projected value corresponds with the actual value, whereas precision pertains to the uniformity of values within the set. The maximum relative error values for R^1^ and R^2^ can be identified from the results illustrated in Fig. 11.

### RNN modeling and performance

The performance indicators were used to evaluate the best RNN models for predicting R1. Among these indicators, RRMSE and MAPE had the highest values of 0.682 and 45.35, respectively, when 3 neurons were used in the hidden layer. On the other hand, the RMSE indicator had the highest value of 39.00 when 8 neurons were employed in the hidden layer. The largest value for the RE indicator was 193.69 when 5 neurons were utilized in the hidden layer. In contrast, the lowest values were observed for all indicators: RRMSE (0.143), MSE (51.63), MAPE (10.11), RMSE (7.18), and RE (43.39). These results are offered in Table 8.

**Table 8:** The performance indicators for RNN models to prediction of R^1^.

Model	Neurons	Structure	RRMSE	MSE	MAPE	RMSE	RE%	R^2^
M1	3	(3–3-1)	0.682	1051.21	45.35	32.42	177.12	0.2499
M2	4	(3–4-1)	0.299	193.06	14.18	13.89	111.03	0.5468
M3	5	(3–5-1)	0.666	955.74	41.54	30.91	193.69	0.0037
M4	6	(3–6-1)	0.143	51.63	10.11	7.18	43.39	0.9002
M5	7	(3–7-1)	0.404	1032.76	36.02	32.13	−60.22	0.0109
M6	8	(3–8-1)	0.475	1521.05	44.61	39.00	−61.13	0.0863
M7	9	(3–9-1)	0.329	586.39	24.47	24.21	−62.5	0.4068
M8	10	(3–10-1)	0.404	302.27	25.20	17.38	100.28	0.2951
M9	11	(3–11-1)	0.237	157.98	18.10	12.56	47.54	0.734
M10	12	(3–12-1)	0.335	625.33	26.39	25.00	−65.75	0.2855

The RRMSE indicator obtained the largest value of 0.458 when 9 neurons were used in the hidden layer. On the other hand, for the indicators MSE, MAPE, RMSE, and RE%, the highest values were observed when 5 neurons were used: 7873.31, 39.54, 88.73, and 100.52, respectively. It is worth noting that all indicators achieved their lowest values with 8 neurons: RRMSE (0.062), MSE (172.08), MAPE (3.850), RMSE (13.11), and RE% (−18.49). These results are presented in Table 9.

**Table 9:** The performance indicators for RNN models to prediction of R^2^.

Model	Neurons	Structure	RRMSE	MSE	MAPE	RMSE	RE%	R^2^
M1	3	(3-3-1)	0.203	2438.08	14.35	49.37	−45.49	0.2826
M2	4	(3-4-1)	0.111	365.43	7.53	19.11	27.36	0.875
M3	5	(3-5-1)	0.446	7873.31	39.54	88.73	100.52	0.1628
M4	6	(3-6-1)	0.162	1053.30	7.77	32.45	−47.32	0.6957
M5	7	(3-7-1)	0.113	1271.06	4.69	35.65	−42.35	0.5717
M6	8	(3-8-1)	0.062	172.08	3.85	13.11	−18.49	0.9471
M7	9	(3-9-1)	0.458	5728.90	35.59	75.68	123.67	0.2282
M8	10	(3-10-1)	0.103	275.67	7.43	16.60	27.22	0.9044
M9	11	(3-11-1)	0.188	1035.15	13.75	32.17	52.15	0.7893
M10	12	(3-12-1)	0.208	1240.00	14.60	35.21	53.50	0.6106

**Figure 12:**
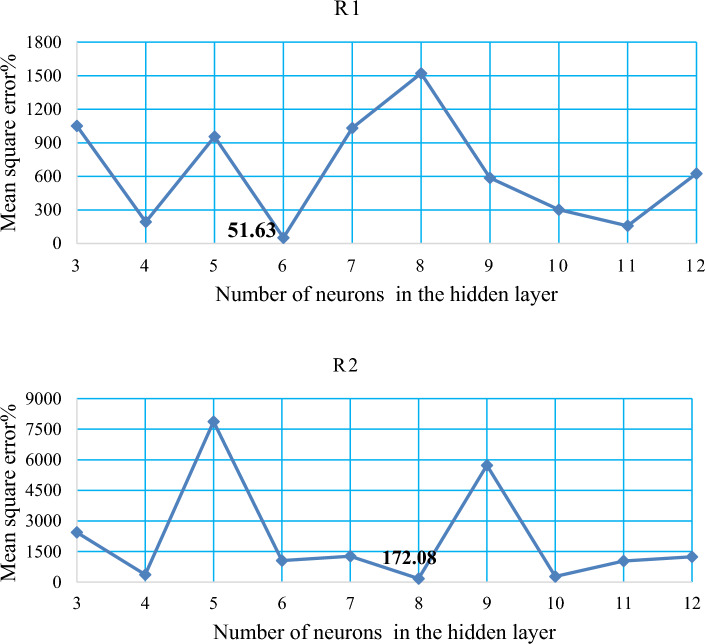
Show the correlation between the number of concealed layer neurons and the MSE obtained to predict (R1, R2).

The MSE value for the network with 3 neurons was found to be 1051.21. However, when the number of neurons increased to 4, the MSE significantly decreased to 193.06. On the other hand, with the application of 5 hidden neurons, the MSE increased to 955.74. Subsequently, when 6 hidden neurons were used, the MSE decreased to 51.63, as depicted in Fig. 12R1. However, with the application of 7 and 8 hidden neurons, the MSE values increased to 1032.76 and 1521.05, respectively. For 9 hidden neurons, the MSE decreased to 586.399, while for 10 hidden neurons, it was 302.27. Furthermore, with 11 hidden neurons, the MSE decreased to 157.98. However, when 12 hidden neurons were utilized, the MSE increased to 625.33.

The MSE value for the network with 3 neurons was found to be 2438.08. However, when the number of neurons increased to 4, the MSE sharply decreased to 365.43. On the other hand, with the application of 5 hidden neurons, the MSE increased to 7873.31. With the addition of another neuron in the hidden layer (6 neurons), the MSE decreased to 1053.30. However, when 7 hidden neurons were used, the MSE increased to 1271.06. Interestingly, with the application of 8 hidden neurons, the MSE sharply decreased to 172.08, as shown in Fig. 12R2. Furthermore, for 9 hidden neurons, the MSE increased to 5728.90. For 10 hidden neurons, the MSE was 275.67. Gradual increment in the MSE was observed when increasing to 11 hidden neurons (MSE = 1035.15). Finally, with the application of 12 hidden neurons, the result of the MSE increased to 1240.00.

**Figure 13:**
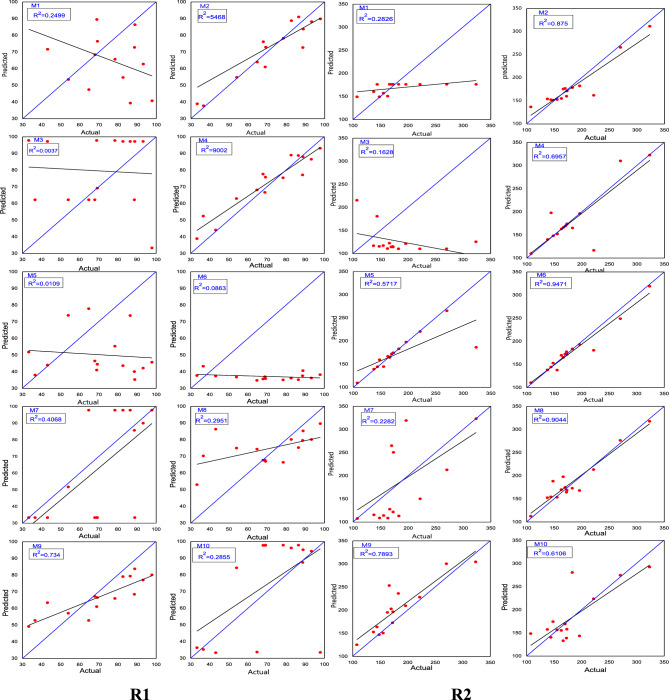
The correlation coefficient between observed and predicted values of R1, R2.

The scatter plots compare the information obtained from the RNN with the experimental data. The best performance in terms of correlation coefficient (R^2^) was achieved when the network structure had 6 hidden neurons in the hidden layer, resulting in an R^2^ value of 0.9002. This model is considered the best for predicting R1, as shown in Fig. 13R1. Similarly, for predicting R^2^, the best performance in terms of correlation coefficient (R^2^) was observed with 8 neurons in the hidden layer, yielding an R^2^ value of 0.9471. This model is considered a better fit for predicting R^2^, as shown in Fig. 13R2.

**Figure 14:**
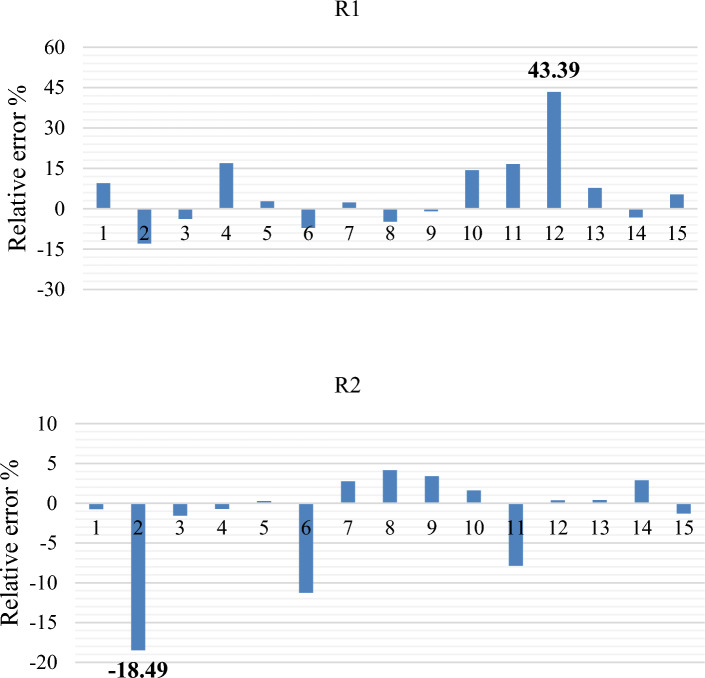
demonstrates the accuracy of the R^1^, R^2^.

Based on the results depicted in Fig. 14R1, the maximum error for R1 is found to be less than 43.39%. Similarly, for R2, the maximum error is less than 18.49%, as revealed in Fig. 14R2. The initial experimentation for FFNN primarily aimed to investigate the number of hidden layer neurons in order to determine the optimal configuration for the network structure. Additionally, it aimed to identify scenarios where the network fails to make accurate predictions. The evaluation metrics MSE and RMSE achieved their lowest values of 14.54 and 3.81, respectively, while obtaining the highest correlation coefficient value of 0.9658 when the hidden layer contained 5 neurons for R1. For R2, the lowest values of MSE and RMSE were observed as 454.60 and 21.32, respectively, along with the best correlation coefficient value of 0.836 when the hidden layer contained 6 neurons. These findings are presented in Table 10.

**Table 10:** The performance indicators for FFNN models to prediction of R1. R2.

FFNN		R1			R2		
Model	Structure	MSE	RMSE	R^2^	MSE	RMSE	R^2^
M3	(3-3-1)	1081.72	32.88	0.061	647.05	25.43	0.776
M4	(3-4-1)	1382.90	37.18	0.032	597.58	24.43	0.786
M5	(3-5-1)	14.54	3.81	0.965	609.55	24.68	0.806
M6	(3-6-1)	703.93	26.53	0.153	454.60	21.32	0.836
M7	(3-7-1)	32.86	5.73	0.929	3200.20	56.57	0.683
M8	(3-8-1)	343.84	18.54	0.254	4720.98	68.70	0.456
M9	(3-9-1)	87.61	9.36	0.787	2574.11	50.73	0.169
M10	(3-10-1)	271.53	16.47	0.547	1414.75	37.61	0.649
M11	(3-11-1)	439.38	20.96	0.203	839.35	28.97	0.731
M12	(3-12-1)	755.14	27.47	0.006	10,770.5	103.78	0.283

In summarizing the modeling performance of both FFNN and RNN models, it can be concluded that the model structure significantly impacts their performance. Fluctuations in error can be observed for both R1 and R2, which can be attributed to the complex relationship between input variables and output. The FFNN model demonstrated better performance with a smaller number of neurons in the hidden layer, while the RNN model required a slightly higher number of neurons to achieve optimal performance. Furthermore, it is evident that increasing the number of neurons negatively affected the performance of both models. This can be attributed to overparameterization and the distribution of weight values when using the SGD optimizer. To enhance the performance of both FFNN and RNN models, it is recommended to employ advanced optimizers instead of SGD optimizer. This can help overcome the computational challenges associated with SGD when increasing the number of neurons.

The second experiment followed a similar approach to the first one, but this time the RNN algorithm was utilized. The results of this experimentation, as presented in Table 11, generally yielded the lowest values for the indicators RMSE and MSE, namely 7.18 and 51.63, respectively. The best value of the correlation coefficient (0.9002) for R^1^ was achieved when 6 neurons were applied in the network structure. Similarly, for R^2^, the lowest values for RMSE and MSE were 13.11 and 172.08, respectively, with the best correlation coefficient value of 0.9471. These results were obtained when the network structure included 8 neurons.

**Table 11:** The performance indicators for RNN models to prediction of R1. R2.

RNN			R1			R2		
Model	Neurons	Structure	MSE	RMSE	R^2^	MSE	RMSE	R^2^
M1	3	(3-3-1)	1051.21	32.42	0.249	2438.08	49.37	0.282
M2	4	(3-4-1)	193.06	13.89	0.546	365.43	19.11	0.87
M3	5	(3-5-1)	955.74	30.91	0.003	7873.31	88.73	0.162
M4	6	(3-6-1)	51.63	7.18	0.900	1053.30	32.45	0.695
M5	7	(3-7-1)	1032.76	32.13	0.010	1271.06	35.65	0.571
M6	8	(3-8-1)	1521.05	39.00	0.086	172.08	13.11	0.947
M7	9	(3-9-1)	586.39	24.21	0.406	5728.90	75.68	0.228
M8	10	(3-10-1)	302.27	17.38	0.295	275.67	16.60	0.904
M9	11	(3-11-1)	157.98	12.56	0.734	1035.15	32.17	0.789
M10	12	(3-12-1)	625.33	25.00	0.285	1240.00	35.21	0.610

## Conclusion

The purpose of this study was to evaluate the efficacy of Feedforward Neural Network (FFNN) and Recurrent Neural Network (RNN) architectures in forecasting Response 1 and Response 2 values. Various performance metrics including RRMSE, MSE, MAPE, RMSE, and RE% were employed to assess the performance of these models. The goal was to minimize these metrics and maximize R^2^ values to identify the optimal model. The results indicated that increasing the number of neurons in the hidden layers had a positive impact on the model's performance. This highlights the significance of selecting an appropriate neuron count for achieving accurate predictions. Moreover, the correlation coefficients between the actual data and predictions served as an indicator of the success of each model. Notably, a model with a correlation coefficient of 0.9002 accurately predicted Response 1, while another model with a correlation coefficient of 0.9471 exhibited outstanding performance in forecasting Response 2. The FFNNs also demonstrated strong performance, achieving a high correlation coefficient value of 0.9658 and a low MSE of 14.54. Therefore, based on this study, it can be concluded that both RNNs and FFNNs are highly capable in data prediction applications, particularly for anticipating Responses 1 and 2. Additionally, valuable insights regarding modeling methodologies have been provided. Once the AI models have demonstrated their high accuracy in prediction, it is recommended for future research to explore the potential of utilizing AI models for input optimization. This can involve identifying the best input variables that maximize the value of removal. Furthermore, future research can explore different neural network topologies or incorporate additional features into the analysis to further enhance the predictive performance.

## Data Availability

The datasets used and/or analysed during the current study are available from the corresponding author upon reasonable request.
